# Progress in Flexible and Wearable Lead-Free Polymer Composites for Radiation Protection

**DOI:** 10.3390/polym16233274

**Published:** 2024-11-25

**Authors:** Shouying Wu, Wei Zhang, Yumin Yang

**Affiliations:** 1 School of Textile and Clothing, Nantong University, Nantong 226019, China; 2 Key Laboratory of Neuroregeneration of Jiangsu and Ministry of Education, Co-Innovation Center of Neuroregeneration, NMPA Key Laboratory for Research and Evaluation of Tissue Engineering Technology Products, Nantong University, Nantong 226001, China

**Keywords:** radiation shielding, flexible and wearable, lead-free, polymer composites, preparation strategies

## Abstract

The rapid development of nuclear technology has brought convenience to medical, industrial, and military fields. However, long-term exposure to a radiation environment with high energy will result in irreversible damage, especially to human health. Traditional lead-based radiation protection materials are heavy, inflexible, inconvenient for applications, and could lead to toxicity hazards and environmental problems. Therefore, it has become a mainstream topic to produce high-performance shielding materials that are lightweight, flexible, and wearable. Polymer composites are less dense and have excellent flexibility and processability, drawing great interest from researchers worldwide. Many attempts have been made to blend functional particles and polymeric matrix to produce flexible and wearable protection composites. This paper presents an extensive overview of the current status of studies on lead-free polymer composites as flexible and wearable protection materials. First, novel functional particles and polymer matrices are discussed, and recent results with potential applications are summarised. In addition, novel strategies for preparing polymeric shielding materials and their respective radiation shielding properties are analyzed. Finally, directions for developing lead-free polymeric shielding materials are indicated, and it is beneficial to provide additional references for obtaining flexible, lightweight, and high-performance wearable shielding materials.

## 1. Introduction

The discovery of ionizing radiation in the 19th century contributed greatly to the understanding of matter and led to the rapid development of medicine, industry, and agriculture [1]. As a pivotal ionizing radiation, X-rays are high-energy photons with high frequency (3 × 10^16^~3 × 10^19^ Hz), extremely short wavelengths (0.01~10 nm), and great energy [2,3], providing high penetrating power on objects. Based on these characteristics, X-rays were extensively utilized in medical diagnosis and therapy, industrial inspection, etc. [4,5,6]. However, X-rays, with their high energy and penetrating power, could damage human DNA, induce cancer and cause a variety of other troubling diseases (e.g., deformities, growth retardation, and impaired brain function) [7,8,9]. Human health would suffer irreversible damage from prolonged exposure to X-rays. As a result, the prevention of ionizing radiation hazards has attracted widespread attention. For instance, to safeguard humanity against potential radiation damages, China has established a corresponding standard (GB 18871-2002) [10] to limit the radiation exposure dose to the human body. The standard stipulates that the exposure dose to the public in China is ≤1mSv/year, and the exposure dose to workers is ≤20 mSv/year [10]. Developing effective strategies for regulating the radiation dosage and minimizing potential risks from ionizing radiation has gradually evolved into a public safety issue.

Increasing the distance from the radiation, controlling the duration of radiation exposure, and applying ionizing radiation shielding materials are three methods to reduce radiation hazards [11,12]. Unfortunately, both increasing the distance from radiation sources and controlling the duration of radiation exposure are not practical for patients and other workers in related occupations [8,13]. In comparison, shielding materials could effectively attenuate the intensity of radiation through the “photoelectric effect”, “compton scattering” or “pair production” [8,14,15], which could reduce or even eliminate the risk to humans from radiation exposure. Therefore, applying shielding materials is considered the most promising method of reducing radiation exposure and protecting human health from radiation hazards. Lead blocks, concrete [16,17,18], and heavy metal oxide glass [19,20,21] are conventional radiation protection materials due to their simplicity of production and outstanding shielding properties [2]. However, the large size, heavy mass, inflexibility, and poor air permeability of traditional block radiation shielding materials make them unable to meet the wearer’s needs. Hence, developing lightweight, flexible, and wearable radiation shielding composites to replace traditional block materials has become an urgent problem.

Polymers and their composites are characterized by light weight, durability, and flexibility, making them suitable as matrices for lightweight and flexible materials [22]. Nowadays, polymer composites with efficient radiation shielding properties have been created by doping the polymer matrix with heavy metal elements (mainly high atomic number elements (high-Z elements)) through physical mixing or chemical reactions. According to their origin, polymer matrices used for radiation shielding materials are divided into two main categories: synthetic polymers and natural polymers. Synthetic polymers mainly involve epoxy resins (EP) [23,24], silicone rubber (SR) [25,26], polyurethane (TPU) [27,28], and polyethylene (PE) [29,30,31]. Synthetic polymers with excellent processability and mechanical properties are popular matrices for radiation shielding materials. For instance, BTO-ER composites prepared with ER as a matrix provided 0.35 mm Pb equivalent attenuation at half of the weight of a 0.35 mm Pb sheet [32]. This lightweight, lead-free, and efficient shielding material demonstrated a practical aspect with great potential. However, prolonged exposure to radiation causes changes in the molecular structure of synthetic polymers, leading to degradation of material properties. In addition, the low interfacial compatibility between the functional particles and the synthetic polymer matrix leads to inhomogeneous dispersion of the functional particles, which seriously affects the radiation shielding properties of the materials. Therefore, many studies are currently devoted to improving stability and interfacial compatibility. Natural polymers including natural leather (NL) [33,34] and cellulose [35,36] have also been verified to be ideal polymer matrices for radiation shielding materials due to their excellent breathability, malleability, and flexibility. Compared with synthetic polymers, natural polymers have excellent chemical reactivity and interfacial compatibility, which enhance the stability and dispersion of functional particles in the matrix and are considered excellent carriers for functional particles. In addition, the excellent air permeability and flexibility make them expected to be promising matrices for wearable radiation shielding materials.

In this review, we classified and analyzed the two core components of radiation shielding materials (functional particles and polymer matrices) and introduced the latest research results with potential applications. In addition, the prospective preparation strategies of wearable and flexible radiation protection materials were categorized, and their advantages and disadvantages were analyzed and discussed. Finally, the challenges of radiation protection materials and potential future development directions were provided, aiming to offer valuable references for the study and fabrication of flexible and wearable radiation shielding materials.

## 2. Performance Parameters of Radiation Protection Materials

Lead equivalent, radiation shielding efficiency (RSE), linear attenuation coefficient (μ), mass attenuation coefficient (μ_m_), half-value layer (HVL), and ten-value layer (TVL) are the basic indexes for evaluating the performance of radiation shielding materials, and these parameters can be tested by an X-ray dosimeter. Lead equivalent is the thickness of the lead layer that achieves the same shielding effect as the specific shielding material. RSE is the ratio of the attenuated X-dose to the initial X-ray dose, and it was calculated by the following Equation (1) [33]:

(1) $$\mathrm{RSE}=\frac{I_{0}-I}{I_{0}}\times 100\%$$

where I_0_ is the initial X-ray dose, and I is the penetrated X-ray dose. The X-ray shielding performance test system consists of an X-ray generator and a dosimeter, and the latter is performed to test the dose of X-rays.

μ is used to describe the attenuation effect of X-rays per unit thickness of material, and μ_m_ is used to describe the attenuation effect of X-rays per unit mass of material, as shown in the following Equations (2) and (3) [8] (d is the thickness of the sample, and ρ is the density of the medium):

(2) $$\mu =-\frac{\ln\frac{I}{I_{0}}}{d}$$

(3) $$\mu_{m}=\frac{\mu }{\rho }$$

HVL, TVL refers to the thickness of protective material required to reduce the intensity of incident X-rays to 50% and 10%, respectively, as shown in the following Equations (4) and (5) [8]:

(4) $$\mathrm{HVL}=\frac{\ln2}{\mu }$$

(5) $$\mathrm{TVL}=\frac{\ln10}{\mu }$$

In addition to the mentioned parameters related to the shielding performance of the radiation material, the moisture permeability, mechanical strength, and thermal stability of the material are also important evaluation indicators. Moisture permeability is tested using a textile water vapor permeability tester. Mechanical strength is assessed by testing the tensile-breaking properties of samples using a tensile strength tester. Thermal stability is tested using TGA.

## 3. New Functional Particles for Radiation Protection

The design goal of radiation protection materials is to maximize the attenuation of radiation, thus effectively reducing the damage caused by radiation. The attenuation of X-rays follows Beer-Lambert law (Equation (6)) [8,37], which attenuates by “photoelectric effect”, “compton scattering” or “pair production”.

(6) $$I=I_{0}\cdot e^{{-d\rho Z}^{4}/{\mathrm{AE}}^{3}}$$

where A is the atomic mass, and E is the photon energy. According to Equation (6), it suggested that high-density and high atomic number elements (high-Z elements) could obtain higher attenuation coefficients, which was in favor of photon energy attenuation. For example, lead (Pb) induces high atomic number (Z = 82) and high density (11.3 g·cm^−3^), showing excellent X-ray attenuation properties. However, lead-containing radiation protection materials have obvious defects, which are mainly manifested in: (1) Elemental lead is toxic [38,39], which could damage the human nervous system, reproductive system, and digestive system, and even cause serious environmental pollution; (2) Lead-containing shielding materials have a “weak absorption region” in the energy range of 40–88 keV for X-rays [40,41], resulting in a single lead-containing shielding material that cannot meet the demand for high-performance radiation protection; (3) Traditional lead-containing materials are large in size, heavy, and inflexibility, which seriously hinders their application in wearable radiation protection clothing. Therefore, developing lightweight, flexible, and wearable lead-free materials for radiation protection has gradually become a mainstream research topic worldwide. Studies have indicated that bismuth (Bi) [42,43,44], tungsten (W) [45,46,47,48], and rare earth (RE) elements (e.g., Gd, Ce, etc.) [49,50,51] exhibited radiation shielding ability due to their high atomic number and density, which expected to reduce or replace the lead in shielding materials. In this section, novel lead-free functional particles are analyzed and discussed.

### 3.1. Bismuth-Based Radiation Protection Materials

Bismuth (Bi) has an atomic number of 83 and a high density of 9.8 g·cm^−3^, similar to lead. In addition, most of its compounds are stable and nontoxic, making it a promising functional particle for radiation protection. As a major bismuth derivative, bismuth oxide (Bi_2_O_3_) has received widespread attention in constructing lead-free radiation protection materials. For example, Alshahri et al. [52] applied Bi_2_O_3_ as a functional filler for low-density polyethylene (LDPE) to prepare Bi_2_O_3_/LDPE composites and investigated their radiation shielding performance. It is suggested that the Bi_2_O_3_ (15%)/LDPE composite attenuated 80% of X-rays (at 47.9 keV). The Bi_2_O_3_ nano-functional filler significantly improved the shielding efficiency of LDPE. The prepared composites are lightweight, economical, and non-toxicity, exhibiting attractiveness in replacing pure lead materials. Muthamma et al. [53] studied the effect of the dimensions of Bi_2_O_3_ functional particles on the shielding property of composites. In the study, Bi_2_O_3_/epoxy composites were prepared using 10 wt% micrometers (~10 μm) or nanometer (~20 nm) scale Bi_2_O_3_ as functional particles, respectively. The results indicated that the shielding properties of the materials are strongly dependent on the size of the functional particles. Compared with the micrometer-sized fillers, the nanoscale functional fillers were more evenly dispersed in the epoxy, facilitating the interaction of the functional particles with the radiation. Thus, the nanocomposites exhibited superior radiation shielding effects and helped to reduce the quality of composites.

**Figure 1:**
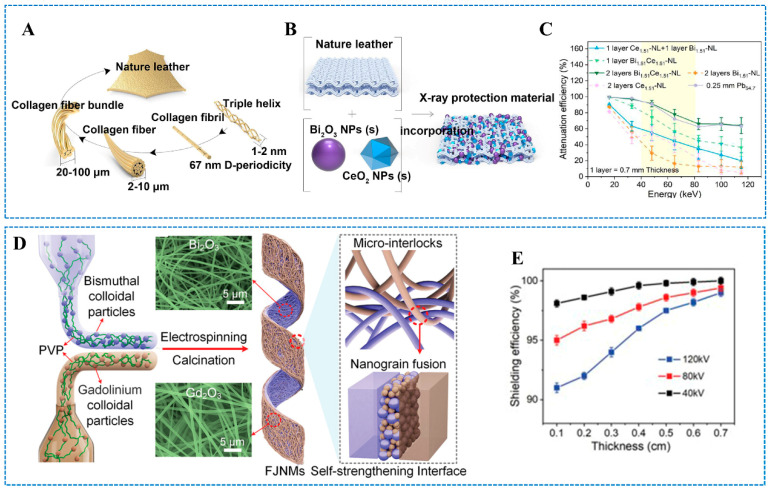
(**A**) Structure of NL. (**B**) Fabrication of Bi/Ce-NL. (**C**) Attenuation efficiency of the Bi/Ce-NL. (**A**–**C**) Reprinted with permission from Ref. [33]. Copyright 2020, Elsevier. (**D**) Structure design of Bi_2_O_3_/Gd_2_O_3_ janus nanofiber membranes. (**E**) Shielding efficiency of Bi_2_O_3_/Gd_2_O_3_ janus nanofiber membranes. (**D**,**E**) Reprinted with permission from Ref. [56]. Copyright 2023, John Wiley and Sons.

The closer the K-shells electron binding energy (i.e., the K absorption edge, or K-edge) of a high-Z atom is to the photon energy, the greater its ability to attenuate the rays [37]. Therefore, when the photon energy decays below the K-absorption edge, the radiation shielding material’s capacity to attenuate photons decreases. Hence, it is difficult for single-component high-Z elements to achieve high shielding efficiency over a wide energy range. To achieve efficient absorption of X-rays over a wide range, a combination of high-Z elements with strong absorption in the weak absorption region of bismuth (<90.5 keV [54,55]) and bismuth is needed to compensate for the absorption blind spot [33]. It is noteworthy that rare earth oxides (Gd_2_O_3_, La_2_O_3_, and CeO_2_) with K-shells electron binding energies ranging from 38.9 to 63.3 keV could recoup the poor absorption area of Bi_2_O_3_ [56]. Thus, Bi_2_O_3_/rare earth oxide composites are expected to be highly efficient X-ray shielding materials due to their complementary X-ray absorption effects. For example, Li et al. [33] reported a novel X-ray shielding material (Bi/Ce-NL) using natural leather loaded with Bi_2_O_3_ and CeO_2_ nanoparticles (Figure 1A–C). The synergistic effect of Bi_2_O_3_ and CeO_2_ nanoparticles significantly contributed to the attenuation of X-rays. When the photons were less than 40 keV, almost 100% of the X-rays were attenuated by the Bi_1.51_Ce_1.51_-NL (1.51 is molecular concentration in NL). In addition, compared with the traditional lead plate, the composite material with low density, excellent physical properties, and water vapor permeability was more suitable for human wear. In addition, to reduce the shielding vulnerability caused by interface bonding defects between functional particles, Xu et al. [56] developed a biphasic Bi_2_O_3_/Gd_2_O_3_ nanofiber membranes (FJNMs) with continuous fibrous structure (Figure 1D,E). The interfacial adhesion of Bi_2_O_3_ and Gd_2_O_3_ was enhanced by the interlocking of nanofibers and the fusion of nanoparticles, which was conducive to the full exploitation of the synergistic absorption of X-ray by Bi_2_O_3_/Gd_2_O_3_. In addition, the prepared fiber membranes exhibit outstanding flexibility, pliability, and permeability because of the flexible combination of Bi_2_O_3_ and Gd_2_O_3_. The flexible combination of Bi_2_O_3_ and rare earth elements is expected to provide more ways for developing highly efficient radiation shielding materials.

In addition to Bi_2_O_3_, other bismuth-containing elemental compounds such as bismuth oxychloride (BiOCl) [57], bismuth vanadate (BiVO_4_) [58], and bismuth titanate (Bi_4_Ti_3_O_12_) [32], have also been tried as functional fillers for radiation protection materials. Based on its environmental friendliness and excellent radiation shielding properties, bismuth is extremely attractive as a replacement material for lead.

### 3.2. Tungsten-Based Radiation Protection Materials

Tungsten (W), with its excellent radiation attenuation based on its high atomic number (Z = 74) and high density (19.3 g·cm^−3^), is another non-toxic potential alternative to elemental lead. A series of composites using tungsten as a functional filler in polymers have been prepared and their effectiveness for radiation protection has been tested.

Monzen et al. [59] prepared a novel paper-based functional material (TFP) based on 80% tungsten powder and 20% cellulose, and seven sheets of TFP paper showed excellent X-ray shielding capability with complete shielding of 50 kVp X-rays. Besides, the cellulose-based TFP material is easy to process and can be folded and cut, exhibiting potential applications as a new flexible and wearable radiation shielding material. In addition, it has been reported that the shielding performance of composites is affected by the size of tungsten and tungsten oxide (WO_3_) functional particles [60,61,62]. For instance, Alavian et al. [62] prepared a new composite material W/LDPE for radiation protection by mixing W and LDPE and analyzed the effect of the size of W particles on the shielding properties of the composite. The results suggested that the attenuation of the rays increases with the decrease in the size of the W particles when the filler ratio is lower, but this size effect is insignificant in higher filler proportions. Furthermore, Elsafi et al. [63] fabricated a novel composite by combining WO_3_ of different sizes (microns/nanoparticles) with epoxy resin to enhance the radiation protection ability of the material at micro and nano scales. Results verified that the homogeneous mixing of microns and nanoparticles in the samples could maximize the density of the epoxy composite. With the composite contained epoxy (50%), micron-WO_3_ (20%), and nano-WO_3_ (30%), its radiation shielding efficiency in the low-energy region (60 keV) was close to 100%, which showed excellent radiation shielding capability. In addition to implementing experimental studies, theoretical predictions and simulations also play an important role in studying the properties of radiation shielding materials, which can improve the reliability of the studies by verifying the accuracy of the experimental data. For example, Özdoğan et al. [64]. prepared a ternary composite radiation shielding material using polyester, barium sulfate, and tungsten and investigated its properties using experiments, theory, and simulations. The results show that the simulation results agree with the experimental results, which proves the reliability of the study results and also emphasizes the feasibility of using simulation to predict the radiation shielding properties of the materials.

### 3.3. Rare Earth Element-Based Radiation Protection Materials

In recent years, rare earth (RE) have exhibited excellent radiation shielding ability due to their unique electronic structure, and fabricating rare earth element-based composites has become a hot topic in radiation shielding. Importantly, the rare earth elements compensate for the weak absorption area of the high-Z elements while also reducing biotoxicity [65,66]. Wang et al. [67] dispersed the La_2_O_3_, Ce_2_O_3_, and Nd_2_O_3_, respectively, into natural rubber (NR) and prepared a series of RE/NR composites. The RE/NR composites have a greater shielding effectiveness for 60 keV photons than the conventional PbO/NR. In addition, Nd_2_O_3_-NR has a thinner thickness and a lighter mass over PbO/NR composites, which is a guideline for developing flexible, lightweight, and lead-free composites. Ogul et al. [68] prepared a radiation protection material using 3D printing by mixing the Gd_2_O_3_ nanoparticles with PLA and explored the potential of the composites. It was shown that 100% shielding efficiency could be achieved using a 10 mm thickness of P-PLA-Gd_20_ polymer, which is expected to be an exceptional alternative to commercial lead aprons.

**Figure 2:**
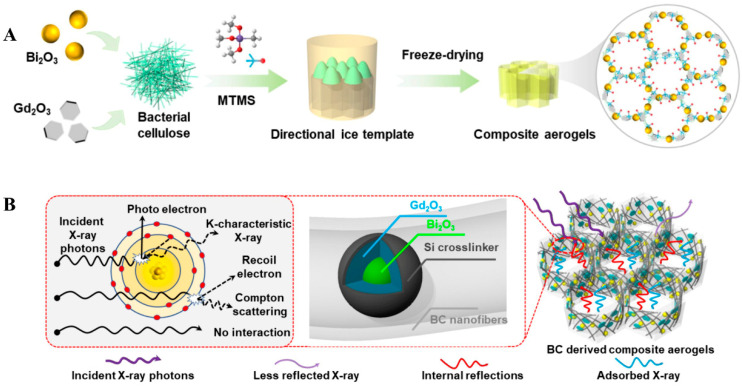
(**A**) Schematic illustration of fabricating BCMBG aerogels. (**B**) Shielding mechanism. (**A**,**B**) Reprinted with permission from Ref. [70]. Copyright 2024, Elsevier.

In addition, the K-edge of rare earth elements increases from 38.9 keV for lanthanum (La) to 63.3 keV for lutetium (Lu), which solved the “weak absorption region” of Pb and other high-Z elements [56,69]. Combinating rare earth elements with other high-Z elements provides a new method to obtain high-performance radiation protection materials. For instance, Sun et al. [70] utilized sustainable nanofibers, Bi_2_O_3_ and Gd_2_O_3_ nanoparticles to design an ultralight and flexible aerogel (BCMBG) (Figure 2). The results showed that the X-ray shielding efficiency was enhanced by multilayer scattering and Bi/Gd elemental absorption synergistically. In detail, the mass attenuation coefficients of the composite up to 6.1 cm^2^·g^−1^, which is equivalent to 3.5 mm Pb-equivalent. In addition, compared with conventional commercial aprons, BCMBG demonstrated ultralightweight, excellent elasticity, anti-pollution, and breathability, which verified its potential for practical applications.

The significant enhancement of shielding efficiency demonstrates the feasibility of rare earth elements as functional particles in enhancing the radiation attenuation capability of materials. In addition, the synergistic effect of rare earth elements and heavy metal oxides enables efficient attenuation of X-rays, providing more possibilities for substituting conventional lead-based materials. However, it is worth noting that the extraction and processing of rare earth elements usually involve high costs, and it might limit the widespread use of rare earth-based shielding materials, and may affect their future competitiveness in the market.

## 4. Polymer Substrates for Radiation Protection

Recently, polymer-based composites have gained a lot of interest and study because of their high flexibility, corrosion resistance, and processability [71,72]. Generally, polymer-based radiation shielding materials are composed of high-Z elements and the polymer matrix. The high-Z elements act as radiation-shielding functional particles uniformly distributed in the polymer matrix through physical adhesion or chemical modification, and the polymer acts as a flexible matrix to load the functional particles. In this section, common polymer materials (e.g., synthetic polymers, natural leather, natural cellulose, etc.) are summarized, and the potential application prospects, challenges, and improvement strategies of these materials are discussed.

### 4.1. Synthetic Polymers

**Figure 3:**
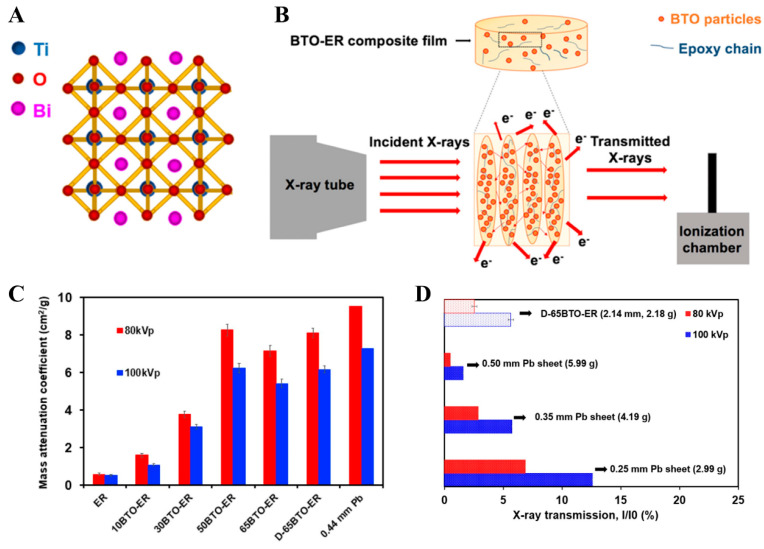
(**A**) Schematic image of BTO. (**B**) The interaction between X-rays and BTO-ER materials. (**C**) Mass attenuation coefficient. (**D**) Comparison of X-ray transmission between pure Pb Film and BTO-ER. (**A**–**D**) Reprinted with permission from Ref. [32]. Copyright 2021, American Chemical Society.

The radiation shielding materials based on synthetic polymers have attracted extensive attention due to their corrosion resistance, high thermal stability, and excellent flexibility and processability [22,49]. Additionally, lightweight and harmlessness to the human body are obvious advantages for synthetic polymers to fabricate wearable radiation shielding materials. Currently, synthetic polymers such as natural rubber (NR) [73], silicone rubber (SR) [74], epoxy resin (EP) [75], polyethylene (PE) [76], poly (lactic acid) (PLA) [77], polystyrene (PS) [78], poly (vinyl alcohol) (PVA) [79], polyester (PET) [80], and polyurethane (TPU) [27] have been utilized for developing flexible and wearable radiation shielding materials. For example, Yu et al. [32] fabricated BTO-ER composites by mixing bismuth titanate (BTO) particles in an epoxy resin matrix and investigated the feasibility of the composites for X-ray shielding (Figure 3). The results suggested that the BTO-ER composites with 65 wt% BTO loading achieved 97% and 95% attenuation efficiencies at 80 kVp and 100 kVp, respectively, with a 2 mm thickness.

However, continuous exposure of synthetic polymers to radiation accelerates organic chain aging, leading to poor durability of polymer composites [2,81]. To solve this issue, researchers have attempted to add other components to the polymer matrix to reduce the cracking of organic chains [82]. Another challenge in constructing high-performance shielding protection materials is the interfacial incompatibility between the matrix and the high-Z elemental nanoparticles [2]. Interfacial incompatibility leads to uneven distribution of functional fillers in the matrix, which ultimately induces structural defects within the matrix and affects the radiation shielding ability and mechanical strength of the composite [2,83,84]. To solve the interfacial problem between the epoxy resin and Gd_2_O_3_, La et al. [85] modified Gd_2_O_3_ with sodium dodecyl sulfate (SDS). The SDS is the surfactant that promotes the homogeneous dispersion of Gd_2_O_3_ in the epoxy resin [2,85]. As a result, the modified Gd_2_O_3_/epoxy nanocomposites exhibited excellent X-ray attenuation efficiency and were even better than the corresponding Pb equivalents at higher energies.

In addition, there is a need to invest more research into hydrophilic modification or suitable structural design of synthetic polymers to develop polymer composites with high comfort and shielding properties.

### 4.2. Natural Leather

Leather is a natural polymer used as a raw material for garments, gloves, and shoes due to its excellent abrasion resistance, flexibility, breathability, and water permeability [86,87]. Compared with synthetic polymers, leather is more advantageous as a substrate for obtaining flexible and wearable radiation shielding materials, mainly reflected in (1) A significant amount of reactive groups (e.g., -COOH, -NH_2_, -OH) contained in the natural leather endow the leather material with excellent chemical reactivity, so it is easier to be loaded with high-Z elements [2,8,34]; (2) The unique three-dimensional fiber network structure of the natural leather facilitates the increase in the interaction between the photons and high-Z elements, promoting multiple scattering and absorption of photons, which could reduce secondary radiation and improve the radiation shielding of the material [88,89]; (3) Natural leather with wear resistance, flexibility, and excellent mechanical strength is beneficial for developing flexible and wearable shielding materials.

**Figure 4:**
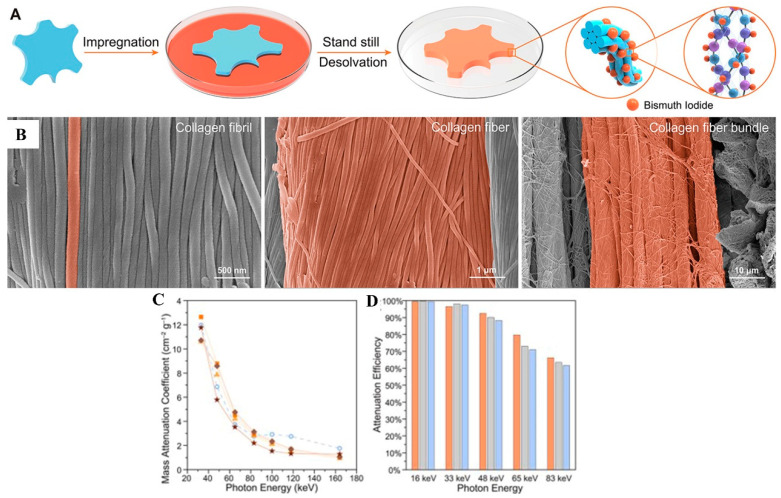
(**A**) The preparation of the BiINP-LM. (**B**) SEM of BiINP-LM. (**C**) Mass attenuation coefficients of BiINP-LM, and shallow circles were 0.25 mm Pb lead apron. (**D**) X-ray shielding performances (orange was 1.00 mm BiINP-LM, gray and blue were lead plate and lead apron, respectively). (**A**–**D**) Reprinted with permission from Ref. [91]. Copyright 2020, American Chemical Society.

The feasibility of using leather as a radiation-shielding material has been investigated. Retanning is an important process in tanning that improves and optimizes the properties of the leather. By adding high-Z elements in the retanning process the radiation-shielding properties of the leather can be improved without damaging the collagen structure of the original leather. Wang et al. [90] fabricated a new radiation-shielding material by loading W (0.35 g·cm^−3^) onto leather through the retanning. It was suggested that the densities of this material were similar to the natural leather. Moreover, due to the effective combination of W and leather, the prepared engineered leather composite material has an attenuation efficiency of up to 100% for photon energy ranges below 40 keV. The unique nanostructure of the leather promotes multiple diffraction and absorption of X-rays and reduces the emission of secondary X-rays, which effectively protects the safety of personnel. In addition, leather-based composites retain the excellent mechanical properties and breathability of virgin leather (10 times higher tensile strength and 3 times higher tear strength than commercial rubber-based materials; more than 300 times higher breathability than commonly used polymers), which makes it a comfortable and safe material for radiation protection. In addition, Wang et al. [91] reported another leather-based composite (BiINP-LM) (Figure 4). In this study, the high-Z elements were stable and well-dispersion in leather because of interactions between BiI and leather. The attenuation efficiency of BiINP-LM with a thickness of 1.00 mm was up to 90% (below 50 keV). In addition, benefit from the leather component the composite exhibited excellent mechanical properties and water vapor permeability, which provides better comfort to the wearer. These outstanding advantages demonstrate the potential of leather-based materials as superior radiation shielding materials.

Compared with traditional lead-based and synthetic polymer-based radiation shielding materials, leather-based composites offer significant advantages, such as abrasion resistance and flexibility, which are important for fabricating lightweight, wearable, and high-performance radiation shielding materials. In addition, the special network structure of leather improved the shielding effect, air permeability, and mechanical properties, which can safely and comfortably protect the human body from the risk of X-ray exposure. Leather is expected to become the advanced radiation shielding material in the future.

### 4.3. Cellulose

Cellulose has been widely used [92,93]. In recent years, to realize the wearability of functional materials, studies have started to apply cellulose fibers as the carrier of functional fillers [94,95,96]. Cellulose contains a large number of hydroxyl groups, thus high-Z elements are stably dispersed on its surface by binding with the hydroxyl groups [2,97]. In addition, cellulose material has a porous structure and complex channels [98], which ensures excellent air permeability and promotes the scattering and dissipation of X-rays inside the material. These properties make cellulose fibers an excellent candidate for fabricating lightweight, soft, and wearable high-performance shielding substrates.

**Figure 5:**
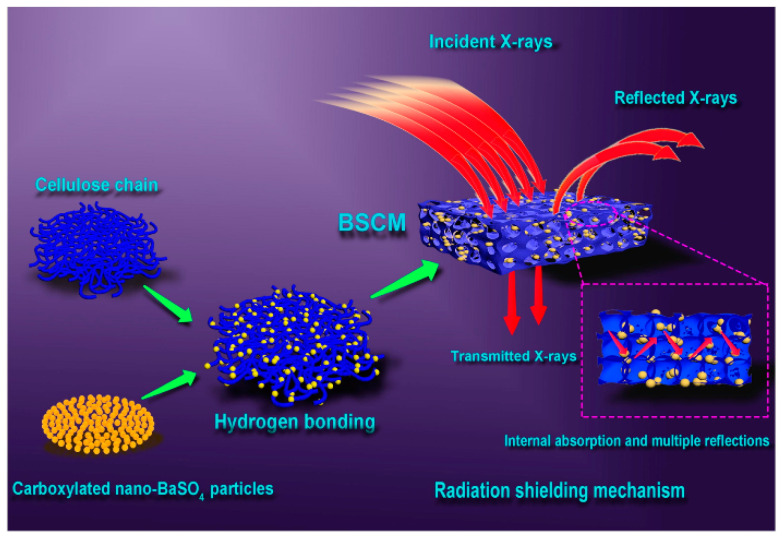
Preparation and shielding mechanism of BSCM. Reprinted with permission from Ref. [35] Copyright 2019, Elsevier.

Jiang et al. [35] designed a novel nanocomposite membrane (BSCM) with a porous and transparent structure using carboxylation-modified nano-BaSO_4_ and fibrous cellulose (Figure 5). The modified nano-BaSO_4_ bonded with the hydroxyl groups of cellulose through hydrogen bonding, which enables uniform and stable dispersion of nano-BaSO_4_ in the matrix, which is beneficial for X-ray attenuation. It was shown that the prepared BSCM composites (containing 20 wt% nano-BaSO_4_) had a high attenuation efficiency of 81.7% for X-rays (at 50 keV).

## 5. Strategies for the Preparation of Radiation Shielding Materials

The preparation methods of shielding materials affect the dispersion of the functional particles and physical structure of the composites. Specifically, uniform dispersion of functional particles in the substrate is a prerequisite for the manufacture of excellent radiation shielding materials, and different structures could cause variations in the permeability and physical properties of the materials. Therefore, the preparation method is crucial for obtaining ideal shielding materials. According to the preparation process, the preparation methods of radiation shielding materials are categorized into coating, spinning, and molding. In this section, common preparation methods are analyzed and summarized, and potential applications of novel preparation strategies are discussed.

### 5.1. Coating Technologies

**Figure 6:**
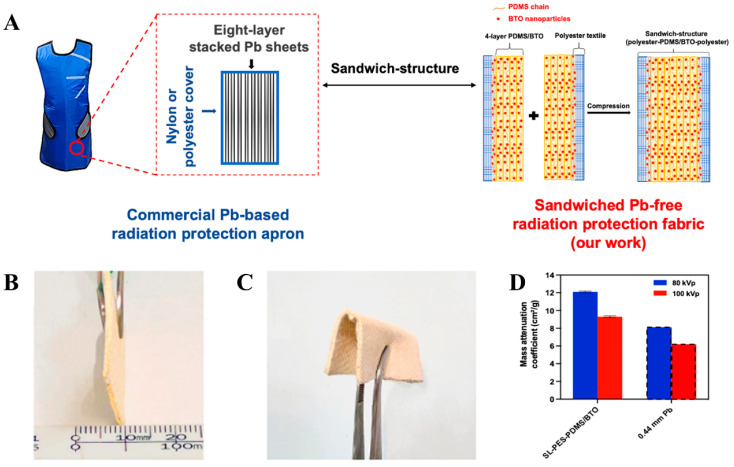
(**A**) Schematic illustration of sandwiched Pb-free radiation protection fabric. (**B**) Thickness of SL-PES-PDMS/BTO. (**C**) Flexibility of SL-PES-PDMS/BTO (**D**) Mass attenuation coefficients of SL-PES-PDMS/BTO. (**A**–**D**) Reprinted with permission from Ref. [104]. Copyright 2022, Elsevier.

The coating technologies mainly uses textiles as the substrates and through scraping, dipping, or sprayingloads the protection functional particles on the surface of the textile to prepare X-ray protection materials [99]. Coating technology can combine functional particles and substrates to obtain the desired radiation shielding material. In addition, the coating can be used as a protective layer for the substrate to prevent degradation of the substrate due to long-term radiation exposure. Due to its flexibility and ease of operation, the coating technique has been used widely [100,101,102]. For example, Maghrabi et al. [103] reported that the mass of Bi_2_O_3_-coated polyester fabrics was reduced by 30% compared with commercial regular lead materials, which solved the problems of heavy mass and poor flexibility of conventional lead materials. One of the advantages of the coating method is its flexibility, which allows the target material to be obtained with different coatings. For instance, Yu et al. [104] prepared a coated fabric with a “sandwich” structure by coating polyester fabric layer by layer with a BTO and polydimethylsiloxane complex (Figure 6). The performance showed that the coated fabric with a thickness of 1.1 mm exhibits a shielding capacity of 0.35 mm Pb equivalent. Besides, this composite material is 42% lighter than a 0.35 mm Pb sheet, which makes this lightweight and flexible textile an excellent alternative to Pb-based composites. Another advantage of coating is the excellent abrasion resistance. Koyuncu et al. [105] reported that the Bi_2_O_3_/WPU-coated fabric (40% w/w WPU and 60% w/w Bi_2_O_3_) possessed excellent X-ray shielding performance and maintained intact appearances after the 50,000 cycles of the abrasion test. However, it is worth noting that the hydrophilicity of the Bi_2_O_3_/WPU-coated fabrics decreased significantly (from 50.05° to 80.12°) compared with that of the uncoated-treated cotton fabrics, which would directly affect the air permeability of the shielding material. In addition, Li et al. [106] reported that the mechanical properties of the material decreased when the content of functional particles in the coating was too high or the coating was too thick.

Coating technology provides a simple and flexible strategy for preparing high-performance flexible and lightweight radiation shielding materials. However, coating technology has some drawbacks, mainly reflected in (1) The functional particles in the coating fall off during use, which in turn affects the protection effect of the coating; (2) Substrate surface with dense coating structure affects the permeability of the protection material, decreasing the protection material applying performance [8]; (3) The thick excessively coating will damage the inherent properties of the fiber, resulting in a decline in mechanical properties.

### 5.2. Spinning Technology

**Figure 7:**
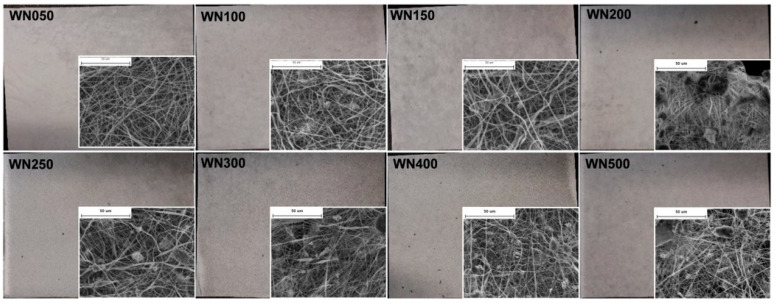
Digital photos and SEM images of W-PU composite nanofiber mats. Reprinted with permission from Ref. [113]. Copyright 2021, John Wiley and Sons.

Spinning, in which X-ray functional particles are dispersed in a polymer solution or melt, and polymer composites are converted into continuous fibers by melt spinning [107,108], solution spinning [109], or electrospinning [110,111]. Nowadays, spinning technology has become a commonly used technique for preparing lightweight and flexible materials. Textiles made of such fibers have a porous structure, so they have excellent air and water permeability, providing wearers with a more comfortable experience [8]. Among them, electrospinning uses electric field forces to stretch charged polymer melts to obtain extremely fine nanofibers. It is currently an effective method to produce radiation protection fibers. For example, Chen et al. [112] fabricated a nanofiber membrane using an electrospinning technique by combing cellulose fluorescent particles, ZnO-Bi_2_O_3_ nanoparticles, and polyacrylonitrile (PAN) solution. The fiber membrane with a dense nanofiber structure provides powerful protection against low-energy X-rays. Yun et al. [113] prepared composite nanofiber mats by electrospinning using tungsten powder and polyurethane (PU) as the original materials (Figure 7), and it indicated that the fiber mats with 400 wt% of tungsten content have a comparable mass attenuation coefficient of 9.64 cm^2^·g^−1^. Moreover, the air permeability and flexibility were 2 and 5 times higher than the conventional film-type sheets, respectively.

Electrospinning textiles offer lower density and more flexibility than normal radiation protection materials, thus being better suited to producing lightweight, soft, and permeable shielding fabrics. However, the materials prepared by electrospinning also have some drawbacks. For example, Hazritz Hazlan et al. [114] reported that nanoparticles with Bi_2_O_3_ content greater than 35 wt% tended to agglomerate in Bi_2_O_3_/PVA composites. In addition, the addition of inorganic functional fillers affects the mechanical properties of the polymer. Therefore, a balance between regulating radiation shielding and maintaining fiber properties is required when using spinning techniques.

### 5.3. Molding Technology

Molding is a technique in which a polymer is shaped by rolling, extruding, or model curing. The molding technology can produce laminated composites with large sizes, which is favorable for scaling up at the industrial level. Li et al. [115] fabricated a multilayer composite (W/ethylene-octene copolymer)/(Bi/ethylene-octene copolymer) using a hot-press method. The X-ray shielding was effectively improved due to the multilayer interfaces and the absorption complementary effect of W/Bi. Zhang et al. [116] used a multilayer co-extrusion technique to produce composites with alternating multilayer structures composed of HDPE/BN and HDPE/BaSO_4_. It verified that the neutron radiation and the secondary radiation generated could be scattered and attenuated by the multilayer interfaces. This alternating multilayer composite exhibits excellent shielding properties due to multilayer interfacial effects compared with conventional polymer blends.

However, composites obtained using the molding method are likely to result in gaps and cracks in the radiation protection material, which affects the radiation shielding performance. In addition, composites prepared by the molding method usually have a multilayer structure, which makes the material less breathable and less flexible, affecting the human wearing experience.

### 5.4. Other Emerging Technologies

To meet the lightweight and comfortable wearing experience and the application requirements of different scenarios, the novel preparation of radiation shielding materials has been explored. Some advanced preparation strategies with potential application prospects have been reported.

Three-dimensional (3D) fabrics prepared by the needling process exhibit excellent air permeability due to high porosity, providing a solution for fabricating comfortable, breathable, and high-performance radiation shielding materials. Accordingly, Wang et al. [107] prepared fibers by melt-spinning 65 wt% of a variety of lead-free functional particles (Bi_2_O_3_, WO_3_, W_2_C, and Ta_2_O_5_) with 35 wt% of polypropylene (PP) into fibers, and then needling was performed to fabricate 3D fabrics. The 3D fabrics possessed excellent radiation shielding efficiency and exhibited lightweight (1/15 and 1/6 of the weight of metallic lead and lead apron, respectively) and excellent air permeability (>1096 mm/s), which has a promising application in X-ray protection.

**Figure 8:**
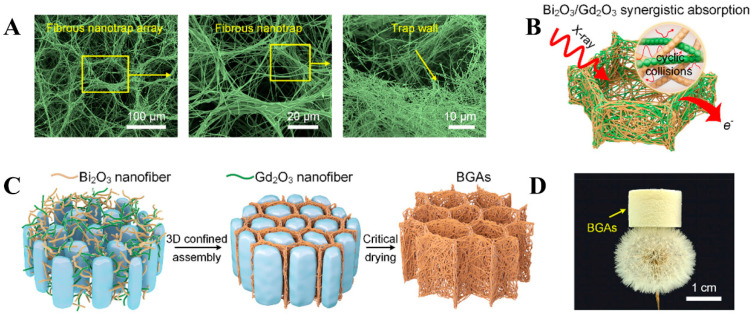
(**A**) Morphologies of BGAs. (**B**) X-ray shielding process of BGAs. (**C**) Fabrication process of the BGAs. (**D**) BGAs standing on a dandelion. (**A**–**D**) Reprinted with permission from Ref. [118]. Copyright 2023, American Chemical Society.

Aerogel materials have a large specific surface area conducive to the adsorption and dispersion of functional particles, and a high porosity that facilitates air exchange, and it was used as flexible and breathable functional textiles [117]. Combinating aerogel materials with functional particles provides new perspectives for preparing radiation shielding materials. To improve the narrow X-ray absorption range, high weight, and poor elasticity of lead-based materials, Xu et al. [118] prepared Bi_2_O_3_/Gd_2_O_3_ aerogels (BGAs) with nanotraps based on cellulose (Figure 8). Bi_2_O_3_, Gd_2_O_3_, and SiO_2_ were first dispersed in water to form nanofiber dispersions, and then silica sol was introduced and homogenized. The BGAs aerogel materials were finally obtained after freeze-drying the dispersions. X-ray energy is effectively attenuated in a wide energy range (16–90 keV) due to the synergistic absorption effect of Bi_2_O_3_/Gd_2_O_3_ and multiple scattering of nanotraps arrays. In addition, the composites exhibit excellent elasticity and breathability, which provides a new strategy for designing next-generation X-ray protection clothing.

3D printing produces solid materials by accumulating materials layer by layer, which provides a new method for manufacturing products with specific shapes and pore structures [119]. As a revolutionary manufacturing method, 3D printing technology has been widely studied in the architectural and medical fields [120,121]. Currently, 3D printing technology has also been attempted to be used for the preparation of radiation shielding materials [122,123]. Compared with other traditional preparation methods, 3D printing technology exhibited greater flexibility and versatility in the structural design of radiation shielding materials, and it will play an increasingly important role in manufacturing shielding materials in the future.

## 6. Conclusions and Outlook

This paper introduces the research progress of wearable flexible lead-free radiation shielding materials, focusing on functional particles, polymer matrices, and composite preparation methods. Compared with traditional lead-based radiation shielding materials, polymer-based composites are lightweight and flexible and show great development potential, practical value, and commercial value in preparing wearable and flexible radiation shielding materials. Table 1 summarizes the characteristics of these novel polymer materials.

However, some challenges still exist:

(1) Permeability: Achieving lightweight and excellent permeability are two important indicators of wearable radiation protection materials. Polymer matrix materials provide a strong guarantee for lightweight, but most synthetic polymers exhibit hydrophobicity, which makes it difficult for water molecules to diffuse from the body to the external environment, seriously affecting comfortableness. Effective strategies for hydrophilic modification of polymers or structural modification of polymer matrix composites need to be developed to enhance the diffusion of water molecules and improve human comfort.
(2) Self-cleaning performance: Radiation shielding materials with self-cleaning capabilities are expected in many cases, especially during medical diagnostic and therapeutic procedures. The self-cleaning radiation shielding materials offer multiple advantages, such as preventing operator exposure to solvents, blood stains, bacteria, and germs, prolonging the life of the material, and reducing post-maintenance costs, etc. The development of protection materials with self-cleaning properties is of great practical significance.
(3) Durability: Shielding materials will undergo chemical changes with time, such as oxidation, hydrolysis, etc., leading to structural damage and performance decline. In addition, with long-term exposure to a high-energy radiation environment, the organic composition of the material will be aging. Hence, material cracks, fractures, and other physical damage occur in the material, which affects the shielding effect and service life. Therefore, there is a need to develop effective methods to improve the durability of polymer composites to ensure that they stably perform their protection for a long period in harsh environments.

## Figures and Tables

**Table 1:** Novel polymer radiation shielding materials and their merits.

Polymer Matrices	Merits
Synthetic polymers matrices (Such as NR, SR, EP, PE, PLA, PS, PVA, PET, TPU, etc.)	(1) Low density ensures that the shielding material is lightweight. (2) High corrosion resistance ensures the durability of the material. (3) Excellent processability to meet the needs of applications of different shapes and sizes.
Natural polymers matrices (Such as leather and cellulose)	(1) Excellent breathability enhances wearer comfort. (2) Abundant chemical groups increase the compatibility of the polymer with the functional particles. (3) Porous structure facilitates radiation attenuation.

## Data Availability

No data was used for the research described in the article.
